# Economic Evaluation of Digital Therapeutic Care Apps for Unsupervised Treatment of Low Back Pain: Monte Carlo Simulation

**DOI:** 10.2196/44585

**Published:** 2023-06-29

**Authors:** Daniel Lewkowicz, Erwin Bottinger, Martin Siegel

**Affiliations:** 1 Digital Health Center Hasso Plattner Insitute University of Potsdam Potsdam Germany; 2 Department of Empirical Health Economics Technische Universität Berlin Berlin Germany

**Keywords:** cost-utility analysis, cost, probabilistic sensitivity analysis, Monte Carlo simulation, low back pain, pain, economic, cost-effectiveness, Markov model, digital therapy, digital health app, mHealth, mobile health, health app, mobile app, orthopedic, QUALY, DALY, quality-adjusted life years, disability-adjusted life years, time horizon, veteran, statistics

## Abstract

**Background:**

Digital therapeutic care (DTC) programs are unsupervised app-based treatments that provide video exercises and educational material to patients with nonspecific low back pain during episodes of pain and functional disability. German statutory health insurance can reimburse DTC programs since 2019, but evidence on efficacy and reasonable pricing remains scarce. This paper presents a probabilistic sensitivity analysis (PSA) to evaluate the efficacy and cost-utility of a DTC app against treatment as usual (TAU) in Germany.

**Objective:**

The aim of this study was to perform a PSA in the form of a Monte Carlo simulation based on the deterministic base case analysis to account for model assumptions and parameter uncertainty. We also intend to explore to what extent the results in this probabilistic analysis differ from the results in the base case analysis and to what extent a shortage of outcome data concerning quality-of-life (QoL) metrics impacts the overall results.

**Methods:**

The PSA builds upon a state-transition Markov chain with a 4-week cycle length over a model time horizon of 3 years from a recently published deterministic cost-utility analysis. A Monte Carlo simulation with 10,000 iterations and a cohort size of 10,000 was employed to evaluate the cost-utility from a societal perspective. Quality-adjusted life years (QALYs) were derived from Veterans RAND 6-Dimension (VR-6D) and Short-Form 6-Dimension (SF-6D) single utility scores. Finally, we also simulated reducing the price for a 3-month app prescription to analyze at which price threshold DTC would result in being the dominant strategy over TAU in Germany.

**Results:**

The Monte Carlo simulation yielded on average a €135.97 (a currency exchange rate of EUR €1=US $1.069 is applicable) incremental cost and 0.004 incremental QALYs per person and year for the unsupervised DTC app strategy compared to in-person physiotherapy in Germany. The corresponding incremental cost-utility ratio (ICUR) amounts to an additional €34,315.19 per additional QALY. DTC yielded more QALYs in 54.96% of the iterations. DTC dominates TAU in 24.04% of the iterations for QALYs. Reducing the app price in the simulation from currently €239.96 to €164.61 for a 3-month prescription could yield a negative ICUR and thus make DTC the dominant strategy, even though the estimated probability of DTC being more effective than TAU is only 54.96%.

**Conclusions:**

Decision-makers should be cautious when considering the reimbursement of DTC apps since no significant treatment effect was found, and the probability of cost-effectiveness remains below 60% even for an infinite willingness-to-pay threshold. More app-based studies involving the utilization of QoL outcome parameters are urgently needed to account for the low and limited precision of the available QoL input parameters, which are crucial to making profound recommendations concerning the cost-utility of novel apps.

## Introduction

### Background

Low back pain (LBP) poses a tremendous health burden for patients and health care systems worldwide, with a lifetime prevalence of up to 85% [1,2]. For patients with nonspecific and nonacute LBP, current clinical guidelines recommend conservative treatment with physiotherapy at regular intervals and increased physical activity [3,4]. Smartphone or web-based digital therapeutic care (DTC) apps offer a novel unsupervised treatment modality for patients with nonspecific LBP [5]. Although DTC apps are now offered by numerous providers, they all follow the same treatment approach, in that video-based exercises aim to replace face-to-face physiotherapy and the provided educational material aims to reinforce patients’ coping abilities for everyday life [5]. A major strength of DTC apps lies in their potential inclusion of decision support interventions, which include tailored push notifications and personalized exercise recommendations that guide subscribed patients through the treatment program [5-7]. These decision support interventions may stimulate persistent engagement and thereby enhance coping abilities and support long-term treatment compliance [8,9].

In Germany, the *Digital Health Care Act* allows statutory health insurance providers to reimburse DTC apps since December 2019, if sound scientific evidence indicates that they are an effective treatment alternative [10]. At present, there are 2 companies, namely ViViRa and HelloBetter, which have developed apps that can provide digital therapeutic via the smartphone or PC and that are now listed in the Digital Health Applications (DiGA) directory to be prescribed for patients with LBP via International Classification of Diseases-10 (ICD-10) code M54 [10]. This paper explores potential trade-offs between higher chances of achieving better long-term health outcomes through lasting behavioral changes, as well as the risk of reimbursing the cost without any benefit for the patients because of higher attrition rates for unsupervised DTC programs as compared to the treatment as usual (TAU; ie, physiotherapy and medication for temporary pain relief [11]).

### Objectives

We applied a probabilistic sensitivity analysis (PSA) to address uncertainties in the transition probabilities, attrition rates, cost components, and health-related quality of life (QoL) scores, which were beyond the scope of the deterministic analysis recently published by Lewkowicz et al [11]. Amending the recently published deterministic analysis offers a relevant contribution to the literature because decision-making based on Markov chains, or other at least moderately complex or nonlinear models, should not be based solely on deterministic models but should include parameter uncertainty as well [12]. Moreover, we intended to explore to what extent the results in this probabilistic analysis differ from the results in the base case analysis and to what extent a shortage of outcome data concerning QOL metrics impacts the overall results. Hence, this underlying PSA intends to reveal the incapacity of a deterministic sensitivity analysis to overcome the challenges of a small patient cohort to simulate the long-term uncertain utility of an intervention. Accordingly, this study aims to inform researchers and decision-makers equally—both to underline the importance of a large data set of QoL data gathered from a large patient cohort and for future approvals of DTC apps for LBP regarding a potential price range, for which such apps may be expected to be a cost-effective alternative to the TAU.

## Methods

### Ethical Considerations

Because this was a simulation study with no human participants, ethics approval was not sought.

### Model Framework

**Figure 1:**
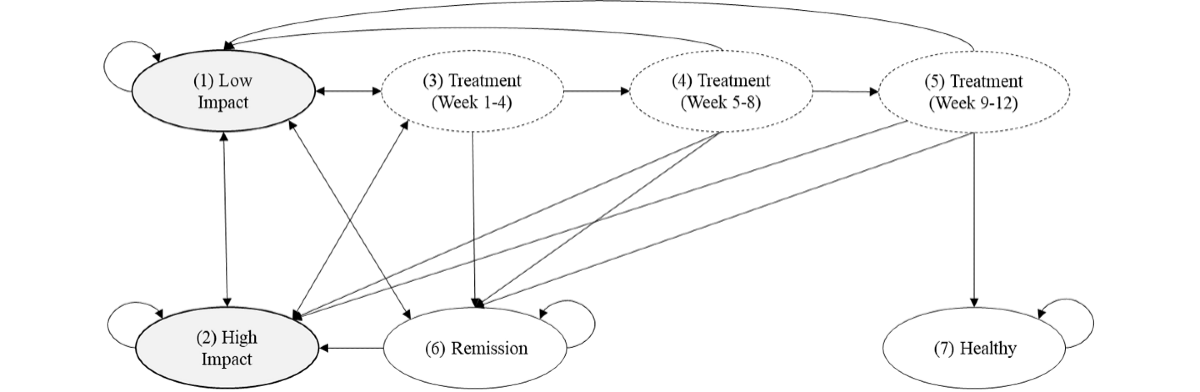
Discrete health state-transition Markov chain with 7 health states (adapted and reprinted from Lewkowicz et al [11]).

This paper builds on a recent analysis of the cost-utility of a DTC program for patients with nonacute LBP in Germany from a societal perspective [11]. The adopted state-transition model in Figure 1 comprises seven distinct health states: (1) low impact of LBP, (2) high impact of LBP, (3) treatment weeks 1 to 4, (4) treatment weeks 5 to 8, (5) treatment weeks 9 to 12, (6) remission, and (7) healthy. States 3, 4, and 5 represent different phases of the treatment progress. State 6 is a state of only temporary improvement, which allows for reoccurring phases with higher or lower pain intensities in the simulation, and state 7 is the final healthy state where no recrudescence can occur.

Like Lewkowicz et al [11], we covered a model time horizon of 3 years and used a cycle length of 4 weeks to allow the inclusion of different treatment states and for patients to drop out before finishing the 3-month course of treatment. Since no published evaluation studies for the ViViRa or HelloBetter DTC apps were available, Lewkowicz et al [11] employed outcome data from an evaluation of the Kaia Health app against 6 face-to-face physiotherapy sessions over a period of 12 weeks [13], arguing that the Kaia Health app is sufficiently similar to the 2 apps currently listed in the DiGA directory.

The transition probabilities for states 3, 4, and 5 were derived from the attrition rates reported in the Kaia Health app study [13]. Patients undergoing app-based treatment continued the program with a chance of 87.5% after each month. In the TAU group, 93.5% of the patients continued the recommended treatment program after the first month, and 95.7% continued after the second month. A recent systematic review on the effects of DTC apps for patients with LBP confirmed this pattern and found that attrition rates can even peak up to 80% in noncontrolled retrospective studies [5].

Lewkowicz et al [11] incorporated several assumptions in their model to be able to specify transition probabilities for their Markov chain. First, the probability of LBP patients visiting a general practitioner, and thus entering treatment, was set to 75% for low-impact LBP and to 80% for high-impact LBP. Second, 50% of the dropout patients were assumed to experience health improvements and thus move to the temporary remission state (state 6). The other 50% of the dropout patients were assumed to have stopped because of coping issues, lack of motivation, or time constraints. Of these, 82.5% fell back into the low-impact LBP state (state 1) and 17.8% fell back into the high-impact LBP state (state 2). Finally, the decision support interventions integrated into the DTC app were assumed to yield a 5% higher chance to transfer to the healthy state (state 7) [8,9,11] than in the TAU strategy [13]. We use the same figures here and display the resulting transition matrices for DTC and TAU in Table 1.

Lewkowicz et al [11] utilized the Veterans RAND 6-Dimension (VR-6D) preference single-utility index [14] derived from the Kaia Health study data [13] for QoLin states 1, 3, 4, and 5. For the remaining states, utility scores based on the Short-Form 6-Dimension (SF-6D) scale were retrieved from other lower back pain (LBP studies [15,16]). The cost components taken from [11] include direct costs for general practitioner and orthopedic consultations, diagnostic procedures, medication, and indirect costs through nonproductive time due to LBP. The price for the DTC app is the current reimbursement price of the ViViRa app of €239.96 for a 3-month prescription (a currency exchange rate of EUR €1=US $1.069 is applicable throughout this paper) [17]. The cost of face-to-face physiotherapy was set to €21.11 per session according to the binding German medical fee schedule [18]. The included utility scores and cost data were discounted with a discount factor of 3% [11].

**Table 1:** Transition matrix of the Markov chain.

To and from			Low impact (state 1)		High impact (state 2)		Treatment weeks 1-4 (state 3)		Treatment weeks 5-8 (state 4)		Treatment weeks 9-12 (state 5)		Remission (state 6)		Healthy (state 7)
**Low impact (state 1)**															
	DTC^a^	0.2		0.0125		0.75^b^		0		0		0.0375		0	
	TAU^c^	0.2		0.0125		0.75^b^		0		0		0.0375		0	
**High impact (state 2)**															
	DTC	0.042		0.158		0.8^b^		0		0		0		0	
	TAU	0.042		0.158		0.8^b^		0		0		0		0	
**Treatment weeks 1-4 (state 3)**															
	DTC	0.0513		0.0111		0		0.87 ^b^		0		0.0625		0	
	TAU	0.0267		0.0057		0		0.935^b^		0		0.0325		0	
**Treatment weeks 5-8 (state 4)**															
	DTC	0.0513		0.0111		0		0		0.875^b^		0.0625		0	
	TAU	0.0176		0.0038		0		0		0.957^b^		0.0215		0	
**Treatment weeks 9-12 (state 5)**															
	DTC	0.2350		0.0509		0		0		0		0.614^b^		0.1^b^	
	TAU	0.2761		0.0598		0		0		0		0.614^b^		0.05^b^	
**Remission (state 6)**															
	DTC	0.5047		0.1092		0		0		0		0.386		0	
	TAU	0.5047		0.1092		0		0		0		0.386		0	
**Healthy (state 7)**															
	DTC	0		0		0		0		0		0		1	
	TAU	0		0		0		0		0		0		1	

^a^DTC: digital therapeutic care.

^b^Transition probabilities taken from the literature. All other transition probabilities in the respective rows are calculated from conditional probabilities given the respective event based on [11].

^c^TAU: treatment as usual.

### PSA Measure

For the PSA, which is a robust method to evaluate the impact of parameter uncertainties [12], we employed the aforementioned model and performed a Monte Carlo simulation with 10,000 iterations. In each iteration, the input parameters were randomly drawn from a priori–defined probability distributions for an entire cohort of 10,000 hypothetical patients. The model time horizon was 3 years with a state length of 4 weeks. We employed a beta distribution to simulate transition probabilities and QoL parameters and a gamma distribution to simulate costs.

We considered the input parameters for transition probabilities and QoL outcomes from the literature as “most likely” values and applied the Program Evaluation and Review Technique (PERT) approximation [19-21] to transform them into estimates for our mean and SD calculations [22] (Multimedia Appendix 1A). We then obtained the shape parameters α and β for the beta distribution through the method of moments [18,21]:



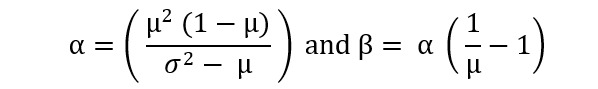



We applied the gamma distribution for all cost components, which requires the mean and SD of the cost components as input parameters. We used the results for direct and indirect cost components of chronic LBP over a 6-month period reported in a large German cost-of-illness study [23] to obtain cost estimates for health states 1, 2, 3, 4, and 5. We assumed costs to be distributed evenly over time and rescale the reported mean costs and the upper and lower limit of the 95% CIs to monthly costs. We derive the SD from the rescaled 95% CIs by dividing the range between the upper and lower limit by twice the 97.5% quantile of the normal distribution [24]:



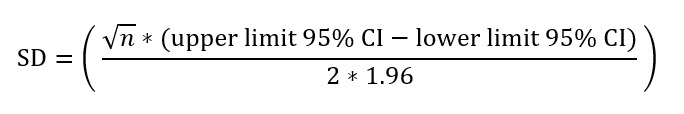



where *n*=51 [23].

We deviated from the assumption in [11] that all physiotherapy costs occur in the first treatment cycle and allocated costs for weekly physiotherapy sessions to states 3 and 4 because they can only be paid if patients continue their treatment. Costs for 4 of the 6 physiotherapy sessions were allocated to state 3, and the remainder was allocated to state 4. The adapted input parameters, including the corresponding distribution parameters, are shown in Tables 2, 3, and 4. Multimedia Appendix 1B contains a full list of all parameters and probability density functions, and Multimedia Appendices 2-5 contain histograms of the parameters and matrices.

We derived cost-effectiveness acceptability curves (CEACs) to illustrate the probability of DTC apps being a cost-effective measure given a certain willingness-to-pay (WTP) threshold. The CEAC indicated the fraction of iterations considered to be cost-effective given a specific WTP. Graphically, the WTP threshold was a line through the origin with a slope equal to the respective WTP, and the outcome of an iteration in the Monte Carlo simulation was considered to be cost-effective if it lies below the WTP threshold in the cost-utility plane [22].

Some health care systems may only adopt novel technologies which are more effective than TAU, (ie, if its incremental effect is nonnegative). We derived an additional CEAC where we included only outcomes that lay in the southeast quadrant or in the northeast quadrant under the WTP threshold in the cost-utility plane to account for this constraint. Moreover, we computed the number of iterations where DTC strictly dominates TAU (ie, where cost__DTC_<cost__TAU_ and effect__DTC_>effect__TAU_, and vice-versa).

**Table 2:** Transition probabilities and beta parameters for simulation after PERT^q^ transformation.

Transition probability			Expected value^b^	SD^c^	α^d^	β^d^
**Temporary health states**						
	**Low impact (state 1) to**					
		Low impact (state 1)	0.30000	0.16667	1.96800	4.59200
		High impact (state 2)	0.02439	0.01234	3.78404	151.36171
		Treatment weeks 1-4 (state 3)	0.66667	0.16667	4.66667	2.33333
		Remission (state 6)	0.06977	0.03612	3.40097	45.34629
	**High impact (state 2) to**					
		Low Impact (state 1)	0.07749	0.04027	3.33817	39.74008
		High Impact (state 2)	0.27200	0.16667	1.66697	4.46160
		Treatment weeks 1-4 (state 3)	0.70000	0.16667	4.59200	1.96800
	**Remission (state 6) to**					
		Low impact (state 1)	0.50314	0.16667	4.02493	3.97471
		High impact (state 2)	0.17938	0.09793	2.57361	11.77401
		Remission (state 6)	0.42400	0.16667	3.30384	4.48823
**DTC^e^**						
	**Treatment weeks 1-4 (state 3) to**					
		Low impact (state 1)	0.09318	0.04880	3.21274	31.26755
		High impact (state 2)	0.02177	0.01100	3.80694	171.09847
		Treatment weeks 5-8 (state 4)	0.80000	0.11024	9.73257	2.43314
		Remission (state 6)	0.11111	0.05871	3.07279	24.58235
	**Treatment weeks 5-8 (state 4) to**					
		Low impact (state 1)	0.09318	0.04880	3.21274	31.26755
		High impact (state 2)	0.02177	0.01100	3.80694	171.09847
		Treatment weeks 9-12 (state 5)	0.80000	0.11024	9.73257	2.43314
		Remission (state 6)	0.11111	0.05871	3.07279	24.58235
	**Treatment weeks 9-12 (state 5) to**					
		Low impact (state 1)	0.32339	0.16667	2.22404	4.65314
		High impact (state 2)	0.09241	0.04838	3.21882	31.61410
		Remission (state 6)	0.57600	0.16667	4.48823	3.30384
		Healthy (state 7)	0.16667	0.09045	2.66255	13.31276
**TAU^f^**						
**Treatment weeks 1-4 (state 3) to**						
		Low impact (state 1)	0.05072	0.02601	3.55883	66.60730
		High impact (state 2)	0.01144	0.00575	3.89784	336.89235
		Treatment weeks 5-8	0.88496	0.06090	23.40459	3.04260
		Remission	0.06103	0.03146	3.47283	53.42822
	**Treatment weeks 5-8 (state 4) to**					
		Low impact (state 1)	0.03414	0.01736	3.69970	104.67097
		High impact (state 2)	0.00760	0.00381	3.93198	513.71610
		Treatment weeks 9-12	0.92081	0.04119	38.65633	3.32444
		Remission (state 6)	0.04123	0.02104	3.63908	84.62987
	**Treatment weeks 9-12 (state 5) to**					
		Low impact (state 1)	0.35079	0.16667	2.52522	4.67334
		High impact (state 2)	0.10684	0.05633	3.10581	25.96486
		Remission (state 6)	0.57600	0.16667	4.48823	3.30384
		Healthy (state 7)	0.09091	0.04756	3.23069	32.30693

^a^PERT: Program Evaluation and Review Technique.

^b^First moment: “Most likely” (expected) value taken from [11].

^c^SD for calculation of the second moment taken from [11].

^d^Shape parameters α and β for beta distribution were calculated using the method of moments.

^e^DTC: digital therapeutic care.

^f^TAU: treatment as usual.

**Table 3:** Cost components.

Cost components (health state)		Mean^a^ (SD^b^)	α^c^	β^c^
Low impact (state 1)		441.74 (476.74)	0.8584	514.5364
High impact (state 2)		588.96 (476.74)	1.5261	385.9023
**Treatment weeks 1-4 (state 3)**				
	GP^d^ consultation	20.47 (43.93)	0.2171	94.2767
	Medication	16.81 (35.36)	0.226	74.3801
	Diagnostic procedure	29.24 (53.72)	0.2962	98.6948
	Indirect cost	147.74 (476.74)	0.0953	1543.6092
	App price (only DTC^e^)	239.96 (N/A^f^)	N/A	N/A
	4 × physiotherapy (only TAU^g^)	102.88 (44.4266)	5.363	19.184
**Treatment weeks 5-8 (state 4)**				
	Medication	16.81 (35.36)	0.226	74.3801
	2 × physiotherapy (only TAU)	46.44 (22.2133)	4.3711	10.6249
**Treatment weeks 9-12 (state 5)**				
	Medication	16.81 (35.36)	0.226	74.3801

^a^Mean values taken from [11].

^b^SD calculated from 95% CIs reported in [23].

^c^Parameters α and β for Gamma distribution calculated from mean and SD values.

^d^GP: general practitioner.

^e^DTC: digital therapeutic care.

^f^N/A: not applicable.

^g^TAU: treatment as usual.

**Table 4:** Health-related QoL^a^ utility scores after PERT^b^ transformation.

Health-related QoL (QALY^c^ weight)		Expected value^d^	SD	α^e^	β^e^
**Health states**					
	Low impact (state 1)	0.655	0.0743^f^	26.1445	13.7708
	High impact (state 2)	0.61	0.1248^g^	8.7032	5.5643
	Remission (state 6)	0.806	0.0713^g^	23.9639	5.7679
	Healthy (state 7)	0.806	0.0713^g^	23.9639	5.7679
**DTC^h^**					
	Treatment weeks 1-4 (state 3)	0.655	0.0766^f^	24.5159	12.9129
	Treatment weeks 5-8 (4)	0.699	0.0695^f^	29.6712	12.7768
	Treatment weeks 9-12 (5)	0.748	0.0699^f^	28.1058	9.4687
**TAU^i^**					
	Treatment weeks 1-4 (3)	0.655	0.0691^f^	30.2894	15.954
	Treatment weeks 5-8 (4)	0.717	0.0834^f^	20.1705	7.9613
	Treatment weeks 9-12 (5)	0.729	0.0862^f^	18.6139	6.9196

^a^QoL: quality of life.

^b^PERT: Program Evaluation and Review Technique.

^c^QALY: quality-adjusted life year.

^d^First moment: “most likely” (expected) value taken from [11].

^e^Shape parameters α and β for beta distribution were calculated using the method of moments.

^f^SD calculated from [13].

^g^SD calculated from [15].

^h^DTC: digital therapeutic care.

^i^TAU: treatment as usual.

## Results

The 10,000 iterations of the Monte Carlo simulation yielded average costs of €2263.96 with an average of 0.6941 QALYs per person and year for DTC and an average cost of €2127.99 with an average of 0.6902 QALYs per person and year for TAU. Thus, the mean incremental cost is €135.97, and the mean incremental QALYs are 0.004 per person and year for the DTC app. The corresponding incremental cost-utility ratio (ICUR) amounts to an additional €34,315.19 per additional QALY. Table 5 shows the summary statistics of the relevant cost and effectiveness outcomes.

**Figure 2:**
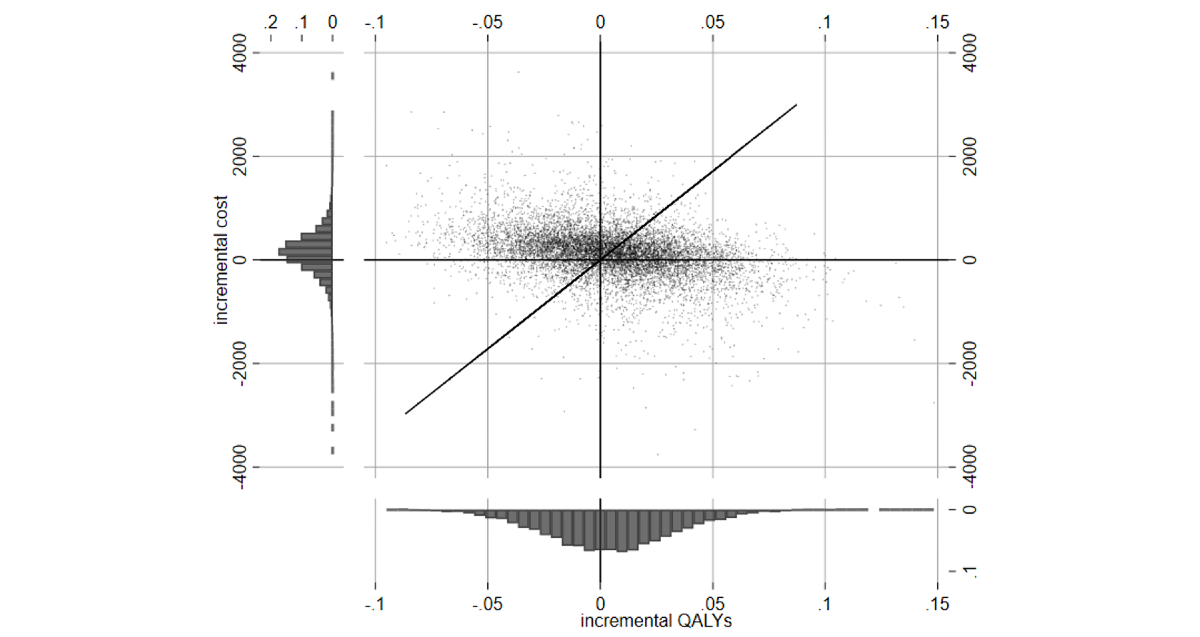
Monte Carlo simulation results per person and year in the cost-utility plane. Each dot represents incremental quality-adjusted life years (QALYs) and incremental costs for one simulated outcome in the cost-utility-plane. Histograms on axes visualize the marginal distributions of incremental costs and incremental QALYs.

Figure 2 shows the simulation results per person and year in the cost-utility plane, where each of the dots reflects 1 outcome of one of the 10,000 Monte Carlo simulations. The histograms on the axes confirm the numbers from the table, which indicate that the mean and median incremental effect, as well as the mean and median incremental cost, are positive. The diagonal line visualizes the estimated average ICUR of 34,315.19.

**Table 5:** Summary statistics of the relevant cost and effectiveness outcomes^a^.

Parameter	Mean (SD)	Median	Min^b^	Max^c^
DTC^d^ cost (€)	2263.96 (1467.69)	1853.92	413.53	22108.91
DTC cost (€) (hypothetical if app price is € 0)	1830.94 (1456.95)	1420.13	99.77	21544.24
DTC QALYs^e^	0.6941 (0.0321)	0.6944	0.5608	0.8223
TAU^f^ cost (€)	2127.99 (1459.20)	1736.76	251.55	21033.72
TAU QALYs	0.6902 (0.0309)	0.6909	0.5711	0.7997
Incremental cost (€)	135.97 (484.54)	149.88	−4748.22	4551.22
Incremental cost (€) (hypothetical, if app price is 0)	1830.9 (1456.9)	1420.1	99.7704	21544.2
Incremental QALYs	0.0040 (0.0296)	0.0038	−0.0950	0.1484

^a^Table shows summary statistics of the simulation results for the regular app price of €239 (a currency exchange rate of EUR €1=US $1.069 is applicable) and the hypothetical scenario with an app price of €0 per person and year.

^b^Min: minimum.

^c^Max: maximum.

^d^DTC: digital therapeutic care.

^e^QALY: quality-adjusted life year.

^f^TAU: treatment as usual.

DTC was costlier than TAU in 66.53% of the iterations but also yielded more QALYs in 54.96% of the iterations. DTC dominated TAU in 24.04% of the iterations, whereas TAU dominated DTC in 35.61% of the iterations. Table 6 gives an overview of the number of iterations, which indicate the different findings.

**Figure 3:**
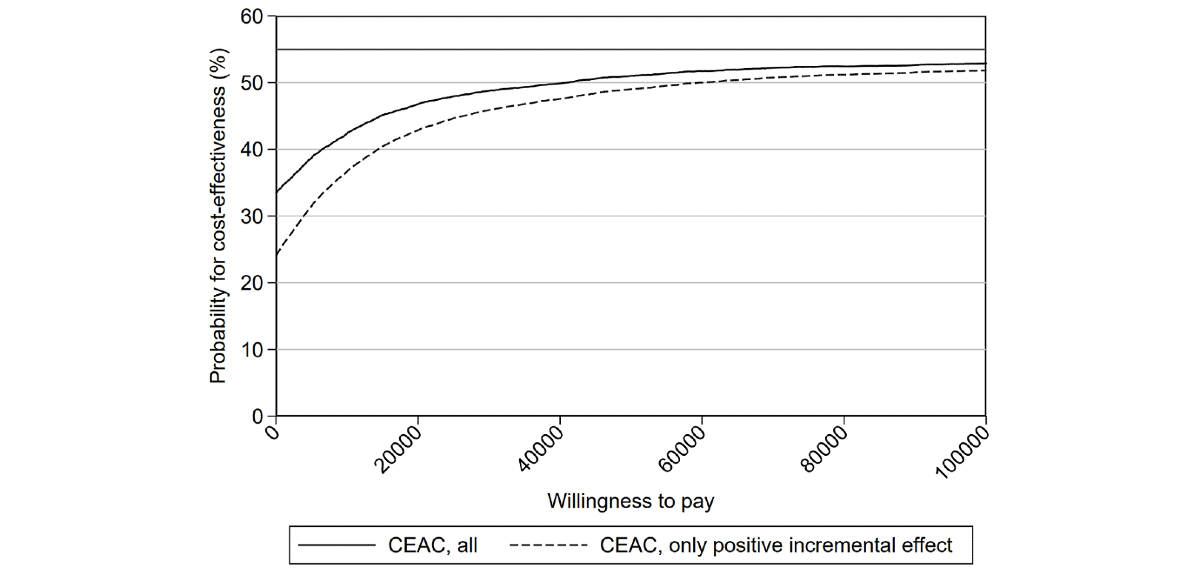
Cost-effectiveness acceptability curve (CEAC) for quality-adjusted life years (QALYs).

The CEAC in Figure 3 illustrates the probability of cost-effectiveness for given WTP thresholds. The solid black line depicts the probability of the DTC strategy being cost-effective given a certain WTP when taking all potential health outcomes into account. The dashed line indicates the probability of DTC being cost-effective at a given WTP under the additional condition that DTC is only acceptable if it produces better health outcomes than TAU. The solid gray line at 54.96% indicates the highest probability of cost-effectiveness at an infinite WTP. Since only 54.96% of the iterations yielded a positive incremental effect and negative incremental effects are unacceptable at an infinite WTP even without the additional condition, both CEACs approximate this threshold.

**Table 6:** Overview of the numbers of iterations, which indicate the different outcomes.

Parameter	Current app price (€239), %	Hypothetical app price (€0), %
Positive incremental treatment outcome	54.96	54.96
Negative incremental treatment outcome	45.04	45.04
Positive incremental cost	66.53	17.64
Negative incremental cost	33.47	82.36
DTC^a^ dominant	24.04	48.85
TAU^b^ dominant	35.61	11.53

^a^DTC: digital therapeutic care.

^b^TAU: treatment as usual.

When including iterations with negative incremental effects, the minimum probability of DTC being effective was 33.47%, corresponding to the fraction of iterations with negative incremental costs. The CEAC reached 50% at a WTP of approximately €41,000, flattened at a WTP of around €80,000, and approximated the maximum possible probability of cost-effectiveness of 54.96% when the WTP tended to infinity. When excluding outcomes with negative incremental effects, DTC was only considered to be cost-effective with a probability of 24.04% for a WTP of €0, corresponding to the fraction of iterations in which DTC strictly dominated TAU. The restricted CEAC reached a probability of cost-effectiveness of 50% only at a WTP of approximately €60,000. Like the unrestricted CEAC, the restricted CEAC flattened around a WTP of €80,000 and approximated the maximum possible probability of cost-effectiveness of 54.96% when WTP tended to infinity.

We reran the Monte Carlo simulation using the same aforementioned figures but with the app cost set to €0 to assess the cost-effectiveness of DTC if the app was available free of charge. Decreasing the app price to €0 yielded a decrease in the incremental cost to €−297.04 and thus a decrease in the ICUR to €−74,964.87. Note that using the same random seed in both simulations assured that the effects and simulated courses of treatment and compliance remained unchanged. Comparing the ICUR with app prices of €239 and €0 allowed us to determine the association between app price and ICUR, which amounts to an increase in the ICUR of €455.41 for each additional Euro charged for a 3-month period. Although the ICUR would be negative for an app price below €164,61, the estimated probability of DTC being more effective than TAU was only 54.96%.

## Discussion

### Principal Findings

This paper presents a PSA to evaluate the potential benefits of an app-based DTC program for patients with LBP in comparison to the TAU in Germany. We found the resulting ICUR to be substantially higher compared to the ICUR in the deterministic base case analysis, indicating that DTC apps are not clearly cost-effective at the current app price of €239 compared to TAU in Germany. The PSA yielded incremental costs of €135.97 and 0.004 incremental QALYs per patient and year for the DTC app. The resulting ICUR was €34,315.19 per QALY gained, as compared to €5,486 reported in [11]. The highest probability of cost-effectiveness for DTC in the PSA was 54.96% at an infinite WTP. Reducing the app price in the simulation from €239.96 to €164.61 for a 3-month prescription could yield a negative ICUR and thus make DTC the dominant strategy, even though the estimated probability of DTC being more effective than TAU is only 54.96%.

The large difference between the ICUR of 34,315.19 found in the PSA and the ICUR of 5,486 reported in [11] can be attributed to the differences in the incremental effects: DTC yielded 0.6941 QALYs per patient and year in the PSA, whereas Lewkowicz et al [11] found 0.697 QALYs per year for DTC. The PSA yielded 0.6902 QALYs per year for TAU, which is similar to the 0.689 QALYs per year reported for TAU in [9].

Overall, the stark difference between the outcome from the PSA and from [11] may be explained by the infinitesimally small incremental effect, indicating that DTC and TAU were similarly effective both in the PSA and in [11]. Since the incremental QALYs appear in the denominator and are close to 0, even small differences may produce drastically different ICURs. With this outcome, a high measurement precision would be required to allow reliable inference from the results, but the available QoL estimate is a single-study outcome derived from 42 participants of the Kaia Health App trial [13], which found no significant difference between DTC and TAU. By including additional states for temporary (state 6) and lasting (state 7) health improvements and simulating a 3-year period, our PSA goes beyond the information available in [13] but still produces similar findings in terms of QoL.

Although a recent review found 12 studies on 6 different DTC apps with implemented decision support interventions, the control groups in those studies received no specific treatment [5]. To the best of our knowledge, the only existing relevant study comparing a DTC app with physiotherapy for our evaluation is the Kaia Health App trial [13], which offered only imprecise estimates for the treatment effect. The limited precision of the available QoL input parameters is reflected in the rather flat histogram of incremental QALYs in Figure 2, which clearly calls for further studies to explore the effects of DTC and decision support interventions on compliance and QoL outcomes for patients with LBP. Particularly, considering that the underlying randomized controlled trial (RCT) [13] only involved a small patient cohort in the app-based intervention group, studies with greater patient cohorts are needed to achieve more precise estimates and to outweigh potential outliers.

The incremental costs of €135.97 found in the PSA are fairly similar to the €121.59 reported in [11]. The primary cost driver in the DTC strategy is the fixed app prescription cost, which occurs every time a patient starts a new treatment program, entering state 3 in the model. These high initial fees may backfire for such highly scalable and easily available app programs, especially if patients’ compliance is unobservable, and there is a high risk for early discontinuation of the DTC. In our simulation, we allowed that the DTC could be prescribed multiple times for 1 patient, which we considered realistic. The higher attrition rate in DTC than in TAU reinforces this major cost driver since the cost of DTC in health state 3 is €239.96 and thus substantially higher than the cost of 4 physiotherapy sessions of €102.88 in the first month. However, it is unclear how often a physician will prescribe the DTC app for the same patient in real life if that patient repeatedly aborts treatment.

Our scenario analysis focused on the effects of the app cost and investigated how the reimbursement price could be updated to render app-based treatment as a cost-effective alternative. The results suggest that an adjusted app reimbursement price less than €164.61, which would be slightly higher than the presumed costs for physiotherapy in the TAU, could lead to negative incremental costs, thus yielding a negative ICUR for the DTC app. Therefore, according to our model, a reimbursement price below €54.87 per month could make DTC somewhat less costly than face-to-face physiotherapy, while the health outcomes cannot be considered to differ significantly between TAU and DTC.

Different DTC programs with different app components and divergently progressed decision support interventions are associated with different overall cost-utility outcomes. While the core components and the core method of health care delivery are similar among these apps, further implementations such as virtual reality guidance during exercises or personalized feedback interventions through push notifications may improve the efficacy of DTC programs and generate increased effects on the QoL of LBP patients. Extended capabilities of decision support interventions may have a significantly positive impact on the long-term outcome [5,9].

To the best of our knowledge, along with [11], this is the first cost-effectiveness analysis for a DTC app based on a RCT for patients with LBP. While we found no clear evidence for a positive incremental effect on health-related QoL but a noticeable increase in cost for the DTC app for LBP, recent studies found DTC apps to be a cost-effective and promising approach for the treatment of unipolar depression [25] and essential hypertension [24].

### Limitations 

The shortage of data may involve potential biases in the parameters of the distributions. We applied the PERT approach to derive probability density functions for the transition probabilities and considered the base-case values from [11] as “most likely” values. However, even though most of the probabilities represent reasonable scenarios in the treatment of LBP, not all parameter values could be derived from clinical findings.

For the gamma distribution, the input values for the standard deviation parameter were derived from a German cost-of-illness study and adopted for the cost components in the PSA. Since we found no information in the literature on potential correlations between different cost components, we sampled each cost component independently in the PSA. The cost outcome may thus be biased either upward or downward, depending on whether higher costs in 1 component increase (eg, if more physician visits trigger more prescriptions) or decrease (eg, if seeing the physician more often avoids costs in other components) the costs in other components. However, since indirect costs make up the largest part of total cost and all cost parameters except for the app reimbursement price and cost of face-to-face physiotherapy are equally included in both strategies, we argue that the missing correlations may have only a relatively small impact on our overall findings.

Our model focused on the direct comparison between the cost of unsupervised DTC and personal physiotherapy, and we excluded inpatient and rehabilitation care, as well as minor ambulatory treatment modalities. Overall, only 81% of total LBP-related health care expenditures were considered in our simulation [23]. It remains unclear what effect an increased use of DTC would have on the utilization of, for instance, injection therapy or surgery. However, we argue that the exclusion of such treatment options does not influence the incremental cost outcome, especially since injection therapy and surgery are usually applied in acute and highly severe cases.

Finally, measuring QoL through 2 different metrics (ie, the SF-6D and VR-6D) is another potential limitation. We acknowledge that using different outcome metrics for 1 simulation is not recommended but argue that SF-6D and VR-6D tend to be highly correlated and yield comparable outcomes, so they may be used interchangeably [14]. Since for both strategies each metric was used similarly for a respective health state, we argue that this methodological choice does not have an impact on the overall results. In addition, probing the results by rerunning the simulation as a cost-effectiveness analysis with pain reduction on a numerical rating scale yielded a similar distribution of the incremental treatment effect (results are available from the authors on request).

### Conclusion

Allowing for parameter uncertainty yielded a significantly higher ICUR than the previously published deterministic approach. The CEACs indicate that the DTC approach is not very likely to be cost-effective, as the probability of cost-effectiveness remains below 55% even for an infinite WTP. One reason for the inconclusive result for QoL may be the high uncertainty, especially in health outcomes. At present, decision-makers should be cautious when considering the reimbursement of DTC apps, since no significant incremental effect on health was found. However, future developments of DTC apps may involve further decision support interventions, which may improve compliance, decrease attrition, and eventually yield better health outcomes. Future evaluations of DTC programs should strive to improve the precision of QoL outcome data and preferably aim to evaluate DTC apps with decision support interventions in a real-life environment.

## Appendix

Multimedia Appendix 1 A. Applying Program Evaluation and Review Technique (PERT) and Method of Moments (MoM) methods. B. Summary of probabilistic sensitivity analysis (PSA) input parameters and probability density functions.

Multimedia Appendix 2 Histogram: cost parameter.

Multimedia Appendix 3 Histogram: quality of life (QoL) parameter.

Multimedia Appendix 4 Histogram: digital therapeutic care (DTC) transition matrix.

Multimedia Appendix 5 Histogram: treatment-as-usual (TAU) transition matrix.

## Abbreviations

CEAC: cost-effectiveness acceptability curve
DiGA: Digital Health Applications
DTC: digital therapeutic care
ICD-10: International Classification of Diseases 10th revision
ICUR: incremental cost-utility ratio
LBP: low back pain
PERT: Program Evaluation and Review Technique
PSA: probabilistic sensitivity analysis
QALY: quality-adjusted life year
QoL: quality of life
RCT: randomized controlled trial
SF-6D: Short-Form 6-Dimension
TAU: treatment as usual
VR-12D: Veterans RAND 12-Item Health Survey
VR-6D: Veterans RAND 6-Dimension
WTP: willingness to pay

## Data Availability

All data generated or analyzed during this study are included in this published paper and its Multimedia Appendix files.
