# Supplementary material for: Economic Evaluation of Digital Therapeutic Care Apps for Unsupervised Treatment of Low Back Pain: Monte Carlo Simulation
Source: JMIR Mhealth Uhealth. 2023 Jun 29;11:e44585. doi: 10.2196/44585 (PMC10365619; doi:10.2196/44585)
Supplement: Multimedia Appendix 1 [file mhealth_v11i1e44585_app1.pdf]

PART 1: Applying the Program Evaluation and Review Technique (PERT) and Method of Moments (MoM).

To calculate the estimated mean and SD from the 'most likely' mode values for the Beta distribution, we utilized the Program Evaluation and Review Technique (PERT) [20]. Accordingly, we adopted and applied the following approximations introduced by Farnum et al. [19]:

If 'most-likely' modal value m < 0.13:

$$\mu = \frac{2}{(2+\frac{1}{m})} \quad \text{and} \quad \sigma = \sqrt{\frac{m^2(1-m)}{(1+m)}}$$

If 'most-likely' modal value $m \in [0.13, 0.87]$:

$$\mu = \frac{4m+1}{6} \quad \text{and} \quad \sigma = \frac{1}{6}$$

If 'most-likely' modal value m > 0.87:

$$\mu = \frac{1}{(3-2m)} \quad \text{and} \quad \sigma = \sqrt{\frac{m(1-m)^2}{(2-m)}}$$

Subsequently, to estimate the shape 1 (α) and shape 2 (β) for the Beta distribution form the new mean μ and SD σ input values, we utilized the Methods of Moments (MoM) [18]. Assuming that a parameter θ follows a beta distribution with shape parameters α and β,

$$\theta \sim \text{beta}\,(\alpha, \beta),$$

the mean μ and variance σ^2 can be used to derive α and β using

$$E\,(\theta) = \frac{\alpha}{\alpha+\beta} = \mu$$

$$\text{var}(\theta) = \frac{\alpha\beta}{(\alpha+\beta)^2(\alpha+\beta+1)} = \sigma^2,$$

such that our estimates for α and β are

$$\alpha = \left(\frac{\mu^2\,(1-\mu)}{\sigma^2 - \mu}\right) \text{ and } \beta = \alpha\left(\frac{1}{\mu} - 1\right).$$

Regarding the Gamma distribution for the cost components, gamma α and β parameters were calculated from the respective mean and SD values with the following formula:

$$\alpha = \frac{\mu^2}{\sigma^2} \quad \text{and} \quad \beta = \frac{\sigma^2}{\mu}$$

PART 2: Summary of PSA input parameters and probability density functions (PDFs)

Digital Therapeutic Care (DTC) strategy

**DTC**	Low Impact	High impact	Treatment week 1-4	Treatment week 4-8	Treatment week 8-12	Remission	Healthy
Low impact	Mean: 0.3<br>SD: 0.1667<br>Mode: 0.2122	Mean: 0.0243<br>SD: 0.0123<br>Mode: 0.018	Mean: 0.667<br>SD: 0.1667<br>Mode: 0.733	0	0	Mean: 0.069<br>SD: 0.036<br>Mode: 0.051	0
High impact	Mean: 0.077<br>SD: 0.0402<br>Mode: 0.0569	Mean: 0.272<br>SD: 0.1667<br>Mode: 0.1615	Mean: 0.7<br>SD: 0.116<br>Mode: 0.7877	0	0	0	0
Treatment week 1-4	Mean: 0.0931<br>SD: 0.0488<br>Mode: 0.0681	Mean: 0.0217<br>SD: 0.011<br>Mode: 0.0162	0	Mean: 0.8<br>SD: 0.11<br>Mode: 0.859	0	Mean: 0.111<br>SD: 0.0587<br>Mode: 0.080	0
Treatment week 4-8	Mean: 0.093<br>SD: 0.0681<br>Mode: 0.0488	Mean: 0.0217<br>SD: 0.011<br>Mode: 0.0162	0	0	Mean: 0.8<br>SD: 0.11<br>Mode: 0.859	Mean: 0.111<br>SD: 0.0587<br>Mode: 0.080	0
Treatment week 8-12	Mean: 0.3233<br>SD: 0.1667<br>Mode: 0.2509	Mean: 0.0924<br>SD: 0.0483<br>Mode: 0.0675	0	0	0	Mean: 0.576<br>SD: 0.1667<br>Mode: 0.602	Mean: 0.1667<br>SD: 0.0904<br>Mode: 0.1189
Remission	Mean: 0.5031<br>SD: 0.1667<br>Mode: 0.504	Mean: 0.1793<br>SD: 0.979<br>Mode: 0.1274	0	0	0	Mean: 0.424<br>SD: 0.1667<br>Mode: 0.3977	0
Healthy	0	0	0	0	0	0	1

DTC Strategy	Low Impact	High impact	Treatment week 1-4	Treatment week 4-8	Treatment week 8-12	Remission	Healthy
Low impact	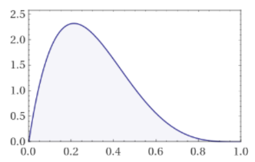	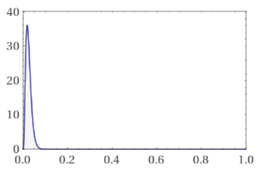	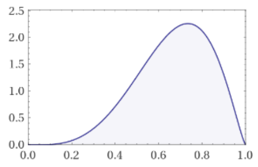	-	-	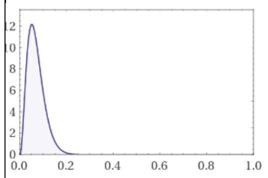	-
High impact	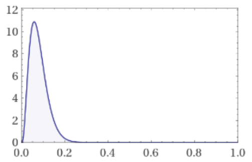	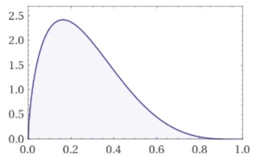	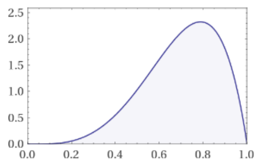	-	-	-	-
Treatment week 1-4	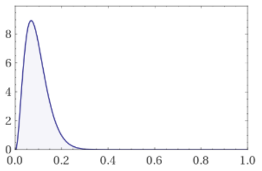	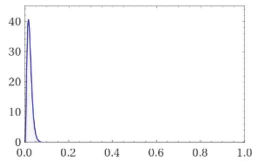	-	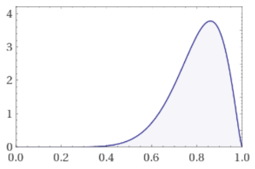	-	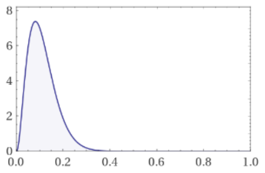	-
Treatment week 4-8	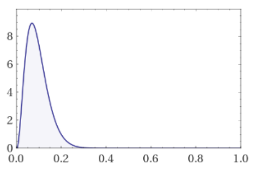	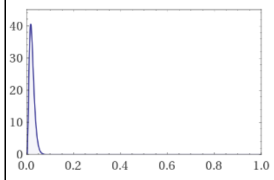	-	-	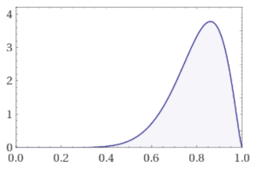	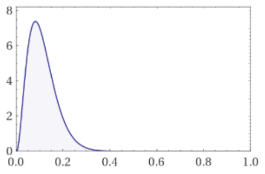	-
Treatment week 8-12	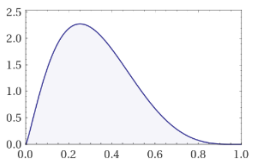	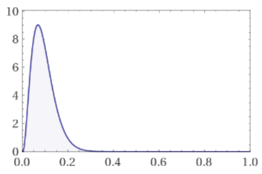	-	-	-	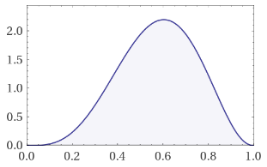	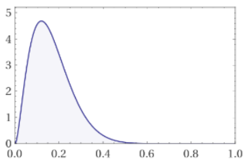
Remission	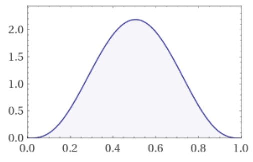	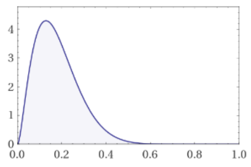	-	-	-	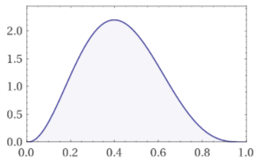	-
Healthy	-	-	-	-	-	-	-

Treatment-as-usual (TAU) strategy

**TAU**	Low Impact	High impact	Treatment week 1-4	Treatment week 4-8	Treatment week 8-12	Remission	Healthy
Low impact	Mean: 0.3<br>SD: 0.1667<br>Mode: 0.2122	Mean: 0.0243<br>SD: 0.0123<br>Mode: 0.018	Mean: 0.667<br>SD: 0.1667<br>Mode: 0.733	0	0	Mean: 0.069<br>SD: 0.036<br>Mode: 0.051	0
High impact	Mean: 0.077<br>SD: 0.0402<br>Mode: 0.0569	Mean: 0.272<br>SD: 0.1667<br>Mode: 0.1615	Mean: 0.7<br>SD: 0.116<br>Mode: 0.7877	0	0	0	0
Treatment week 1-4	Mean: 0.0507<br>SD: 0.026<br>Mode: 0.0375	Mean: 0.0114<br>SD: 0.0057<br>Mode: 0.0085	0	Mean: 0.884<br>SD: 0.0609<br>Mode: 0.9164	0	Mean: 0.061<br>SD: 0.0314<br>Mode: 0.045	0
Treatment week 4-8	Mean: 0.0341<br>SD: 0.0173<br>Mode: 0.0253	Mean: 0.0075<br>SD: 0.0038<br>Mode: 0.0056	0	0	Mean: 0.9208<br>SD: 0.0411<br>Mode: 0.9418	Mean: 0.0412<br>SD: 0.021<br>Mode: 0.0305	0
Treatment week 8-12	Mean: 0.3507<br>SD: 0.1667<br>Mode: 0.2933	Mean: 0.1068<br>SD: 0.0563<br>Mode. 0.077	0	0	0	Mean: 0.576<br>SD: 0.1667<br>Mode: 0.602	Mean: 0.0909<br>SD: 0.0475<br>Mode: 0.0665
Remission	Mean: 0.5031<br>SD: 0.1667<br>Mode: 0.504	Mean: 0.1793<br>SD: 0.979<br>Mode: 0.1274	0	0	0	Mean: 0.424<br>SD: 0.1667<br>Mode: 0.3977	0
Healthy	0	0	0	0	0	0	1

**TAU Strategy**	Low Impact	High impact	Treatment week 1-4	Treatment week 4-8	Treatment week 8-12	Remission	Healthy
Low impact	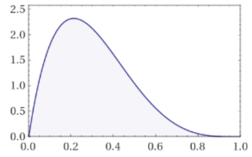	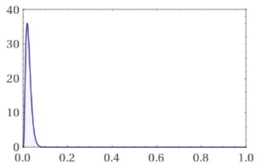	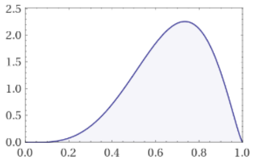	-	-	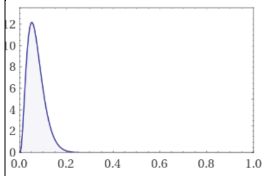	-
High impact	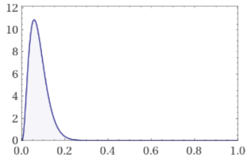	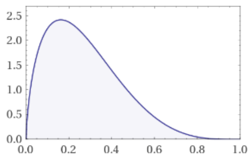	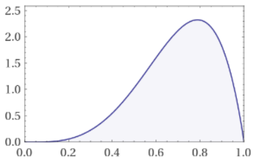	-	-	-	-
Treatment week 1-4	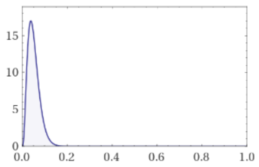	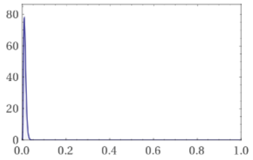	-	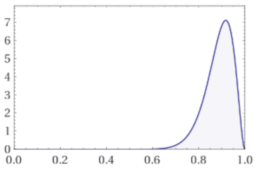	-	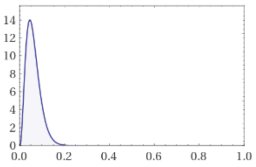	-
Treatment week 4-8	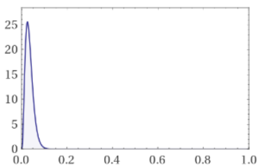	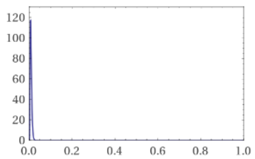	-	-	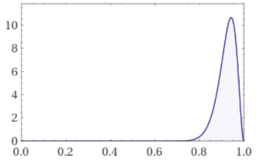	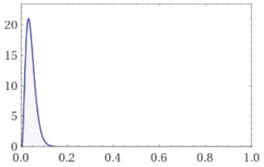	-
Treatment week 8-12	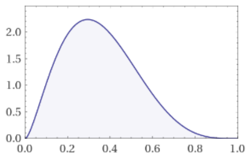	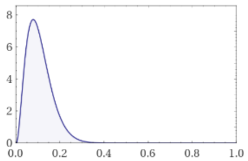	-	-	-	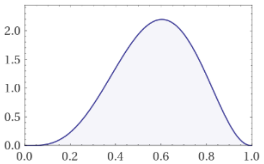	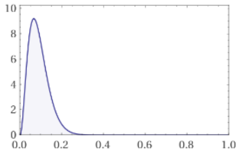
Remission	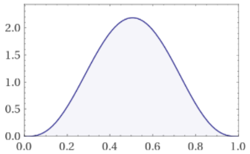	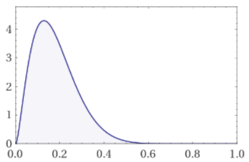	-	-	-	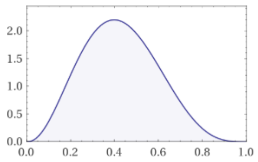	-
Healthy	-	-	-	-	-	-	-

QoL parameters – input values and PDFs

QoL values per health state	DTC	TAU
Low impact	Mean: 0.655, SD: 0.0743, Mode: 0.6631 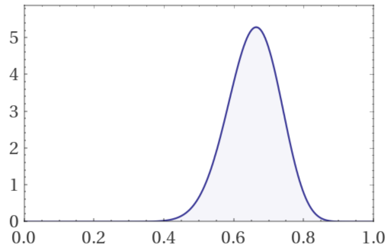	
High impact	Mean: 0.61, SD: 0.1248, Mode: 0.627 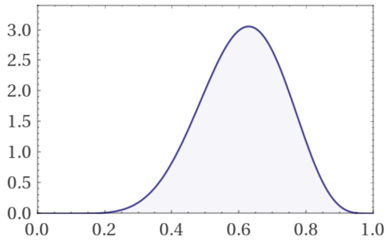	
Treatment week 1-4	Mean: 0.655, SD: 0.0766, Mode: 0.663 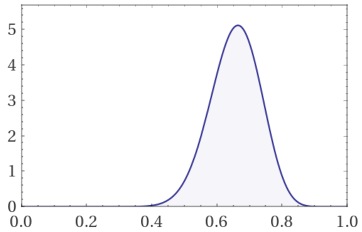	Mean: 0.655, SD: 0.0691, Mode: 0.662 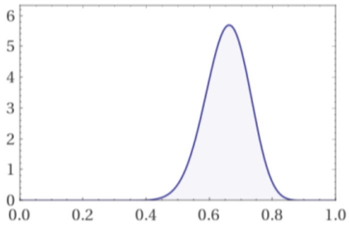
Treatment week 4-8	Mean: 0.699, SD: 0.069, Mode: 0.708 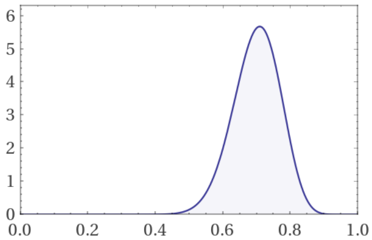	Mean: 0.717, SD: 0.0834, Mode: 0.733 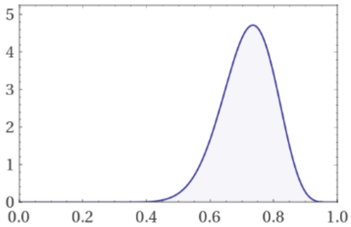
Treatment week 8-12	Mean: 0.748, SD: 0.0699, Mode: 0.762 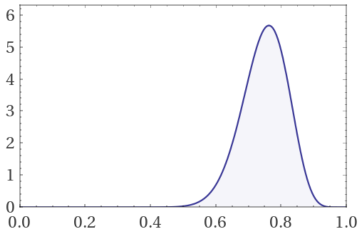	Mean: 0.729, SD: 0.0862, Mode: 0.748 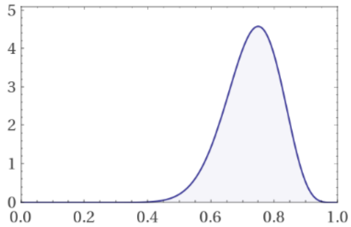
Remission<br>----<br>Healthy	Mean: 0.806, SD: 0.0713, Mode: 0.828 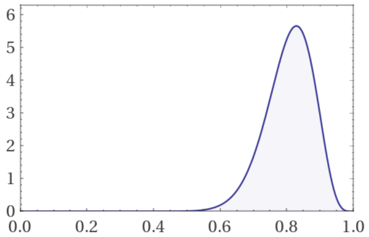	

Cost components – input values and PDFs

Health states / cost components	Mean
Low Impact	Mean: 441.74, SD: 476.74, Mode: (none)<br>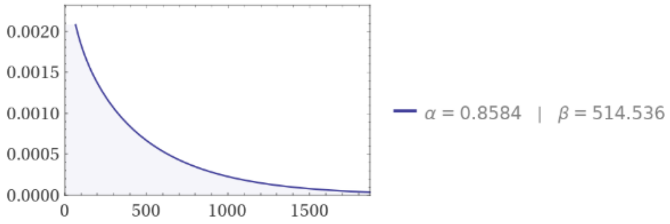
High Impact	Mean: 588.96, SD: 476.74, Mode: 203.02<br>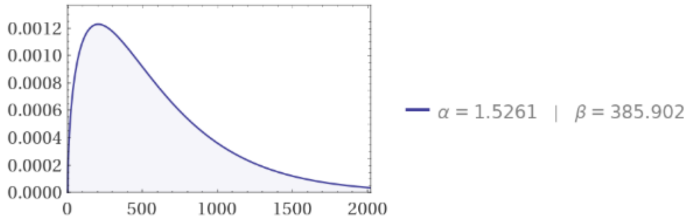
Treatment week 1-4	
GP consultation	Mean: 20.47, SD: 43.93, Mode: (none)<br>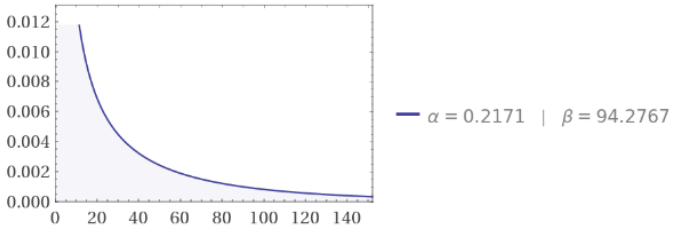
Medication	Mean: 16.8, SD: 35.36, Mode: (none)<br>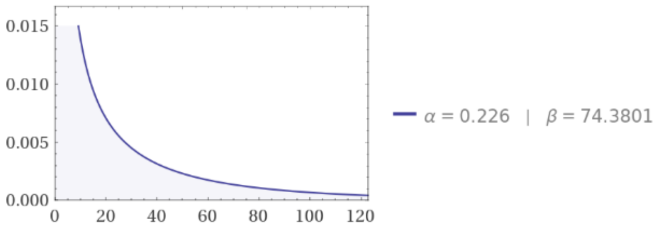
Diagnostic procedure	Mean: 29.24, SD: 53.72, Mode: (none)<br>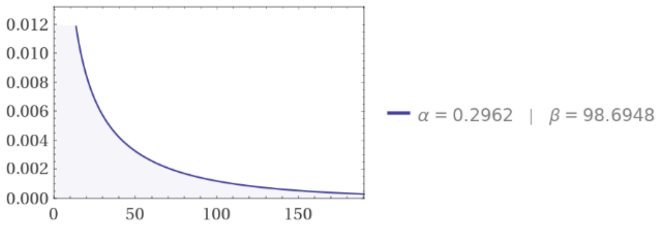
Indirect cost	Mean: 147.74, SD: 476.74, Mode: (none)<br>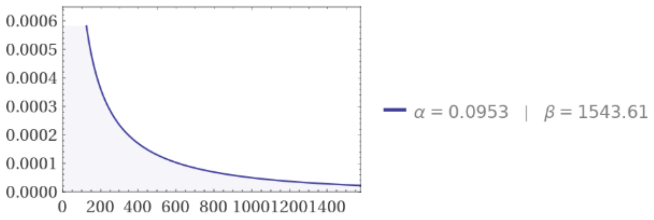

TAU: Physiotherapy (4x sessions)	Mean: 102.88, SD: 44.426, Mode: 83.69 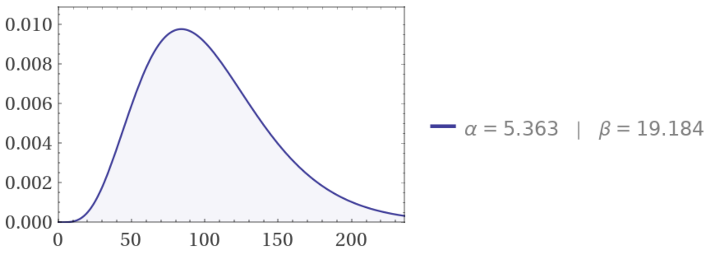
TAU: Physiotherapy (2x sessions)	Mean: 46.44, SD: 22.213, Mode: 35.81 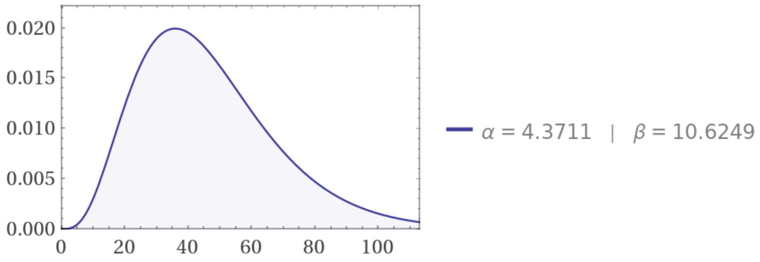

All probability density functions (PDFs) in this document were plotted using Wolfram Alpha computational intelligence. Reference: Wolfram Alpha LLC. 2009. Wolfram | Alpha. https://www.wolframalpha.com/ [accessed on 27.03.2022].

Explanation why n=451 more realistic than n=10.000 for SD calculation in Gamma distribution

<u>Example: Indirect cost (daily wage **mean = €147.24**)</u>

With N= 451	With N= 10.000
**SD: 476.74**<br><br>alpha= 0.095386834, beta= 1543.609261	**SD: 2,244.90**<br><br>alpha= 0.004301873, beta= 34226.94927
PDF:<br>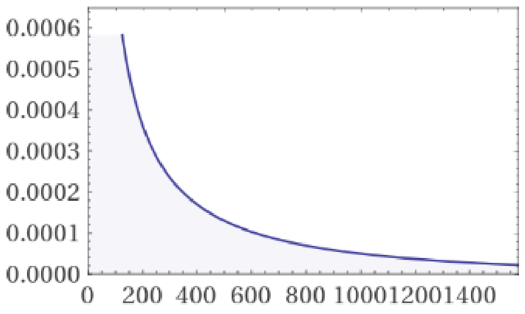	PDF:<br>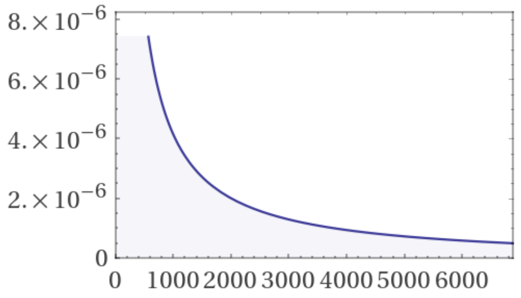
CDF:<br>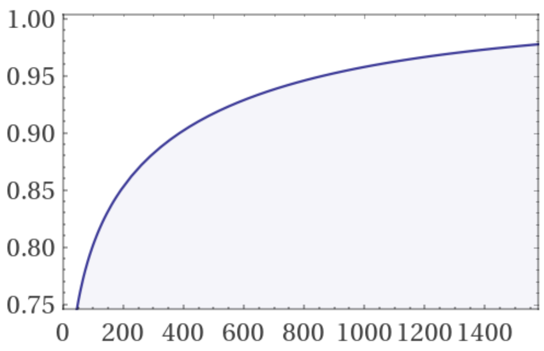	CDF:<br>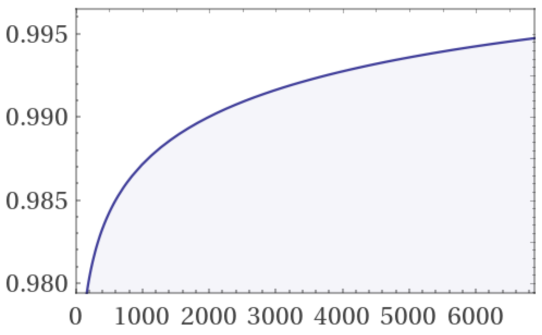
Random example values:<br><br>353.045 | 24.2523 | 1.30707 | 19.3531 | 3286.07	Random example values:<br><br>3.46906×10^-162 | 3.02877×10^-13 | 1.54951×10^-65 | 4.90278×10^-27 | 1.82347×10^-49

Beta distribution parameter for measured health effect:

Numeric Rating Scores (NRS) linearly rescaled to [0;1]

Health states	DTC	TAU
Low impact	Mean: 0.538, SD: 0.17, Mode: 0.551.<br>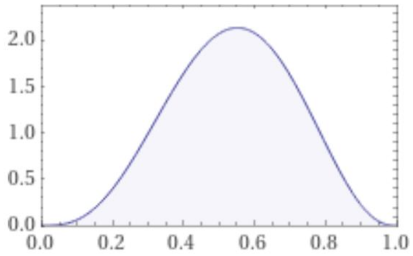	
High impact	Mean: 0.64, SD: 0.24, Mode: 0.92<br>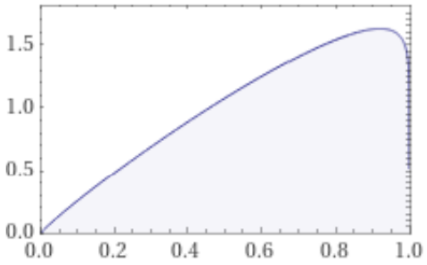	
Treatment week 1-4	Mean: 0.525, SD: 0.107, Mode: 0.527<br>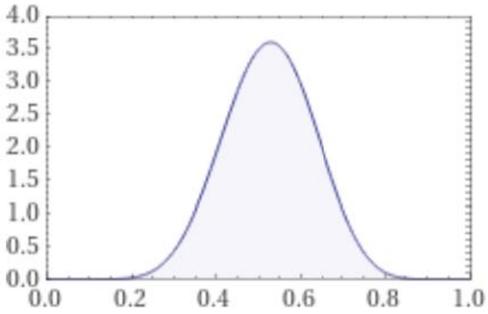	Mean: 0.525, SD: 0.107, Mode: 0.527<br>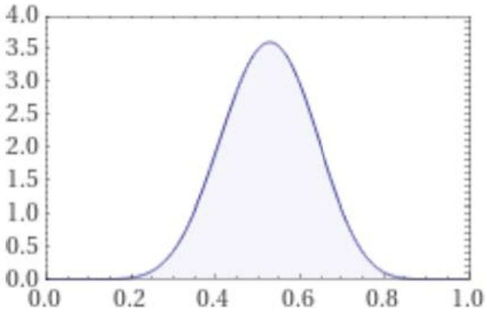
Treatment week 4-8	Mean: 0.439, SD: 0.111, Mode: 0.4318<br>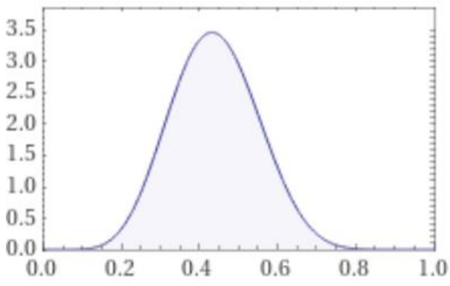	Mean: 0.403, SD: 0.142, Mode: 0.3812<br>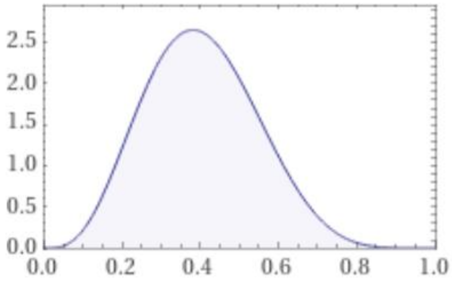
Treatment week 8-12	Mean: 0.27, SD: 0.151, Mode: 0.1885<br>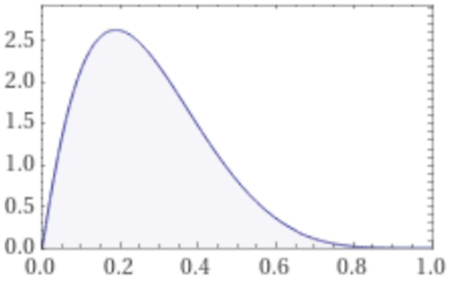	Mean: 0.34, SD: 0.163, Mode: 0.2812<br>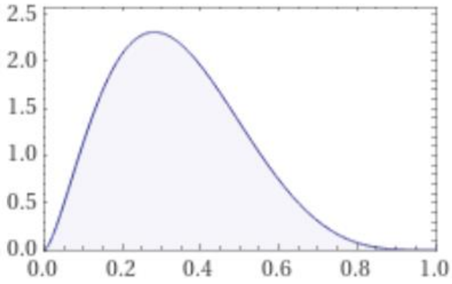
Remission & Healthy	Mean: 0.24, SD: 0.23, Mode: -	

	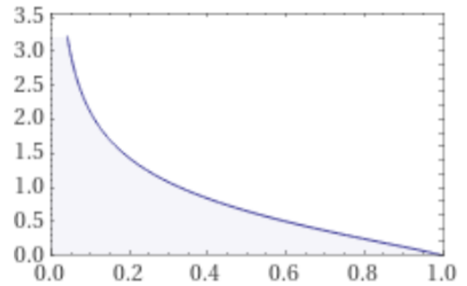 3.5 3.0 2.5 2.0 1.5 1.0 0.5 0.0 0.0 0.2 0.4 0.6 0.8 1.0
