# Supplementary figures and images for: Economic Evaluation of Digital Therapeutic Care Apps for Unsupervised Treatment of Low Back Pain: Monte Carlo Simulation
Source: JMIR Mhealth Uhealth. 2023 Jun 29;11:e44585. doi: 10.2196/44585 (PMC10365619; doi:10.2196/44585)

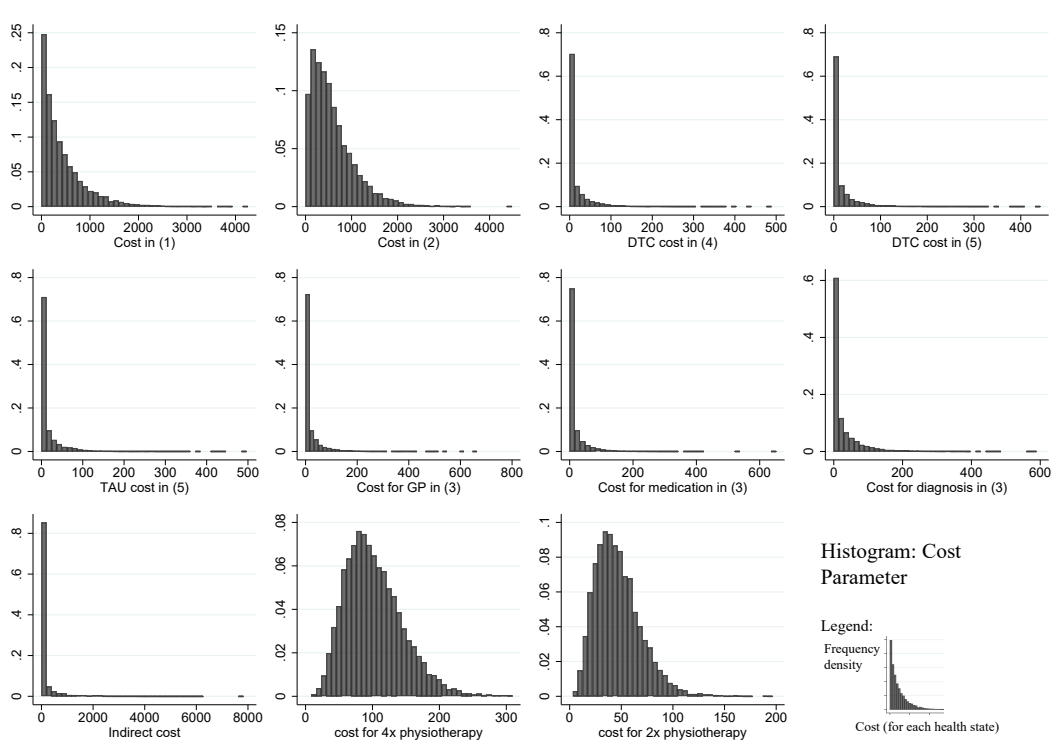

Supplement: Multimedia Appendix 2 [file mhealth_v11i1e44585_app2.pdf]

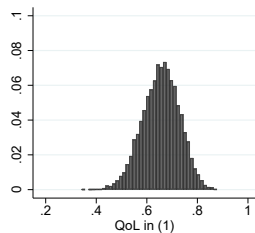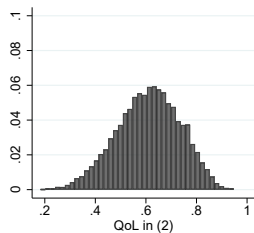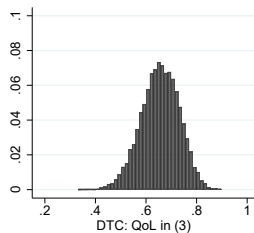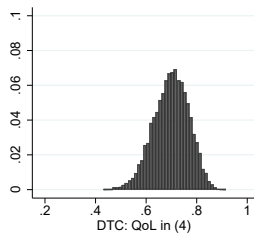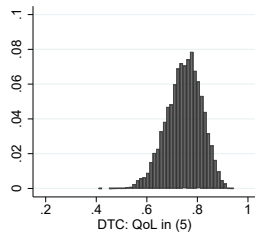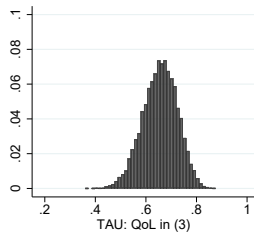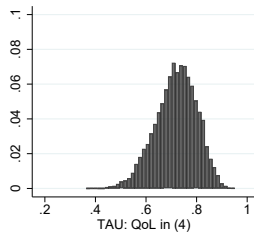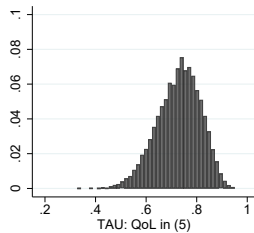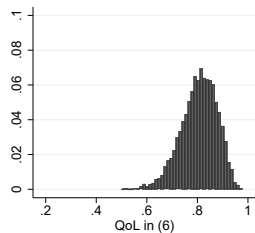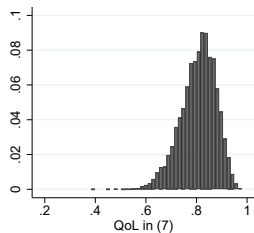

## Histogram: QoL Parameter

Legend:  
Frequency density

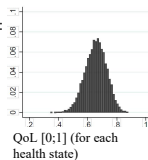

Supplement: Multimedia Appendix 3 [file mhealth_v11i1e44585_app3.pdf]

# Histogram: DTC transition parameter

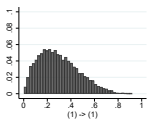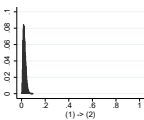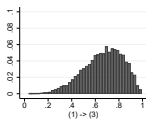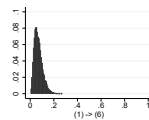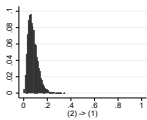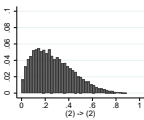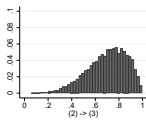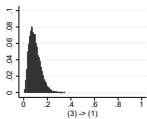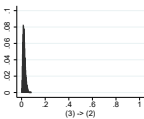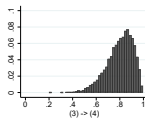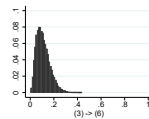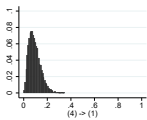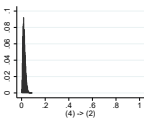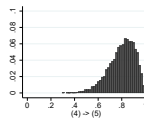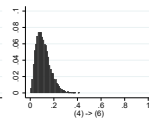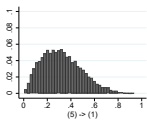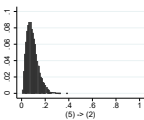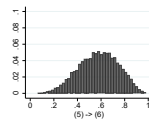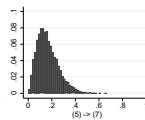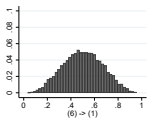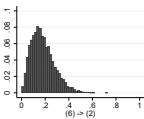

## Legend:

Frequency density

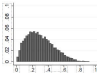

Transition probability

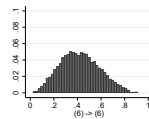

Supplement: Multimedia Appendix 4 [file mhealth_v11i1e44585_app4.pdf]

Histogram:  
TAU transition parameter

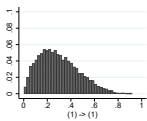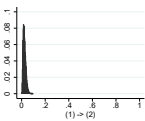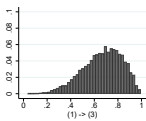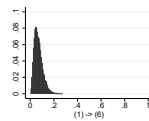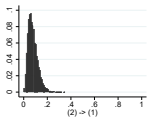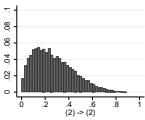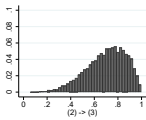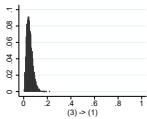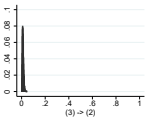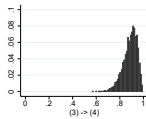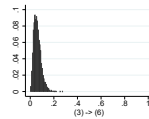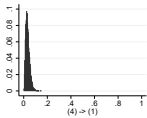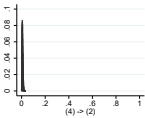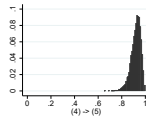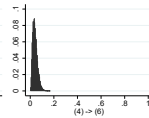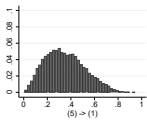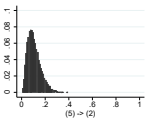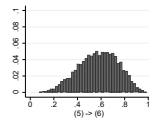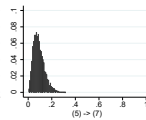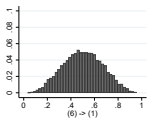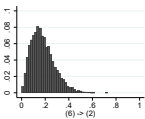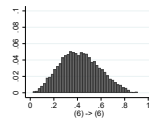

Legend:

Frequency  
density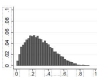

Transition probability

Supplement: Multimedia Appendix 5 [file mhealth_v11i1e44585_app5.pdf]
